# Temporal Molecular Signatures of Early Human Clavicle Fracture Healing: Characterization of Hematological, Cytokine, and miRNA Profiles

**DOI:** 10.3390/ijms26188825

**Published:** 2025-09-10

**Authors:** Li Wan, Sandra Failer, Nadja Muehlhaupt, Christina Schwenk, Peter Biberthaler, Conrad Ketzer, Gregor Roemmermann, Olivia Bohe, Marc Hanschen

**Affiliations:** 1 Experimental Trauma Surgery, TUM Klinikum, Rechts der Isar, School of Medicine and Health, Technical University of Munich, 81675 Munich, Germany; li.wan@tum.de (L.W.);; 2 Department of Trauma Surgery, TUM Klinikum, Rechts der Isar, School of Medicine and Health, Technical University of Munich, 81675 Munich, Germany

**Keywords:** fracture healing, microRNAs, cytokines, fracture severity, temporal dynamics, molecular biomarkers, bone repair

## Abstract

Fracture healing failure affects millions globally, yet early molecular mechanisms remain poorly understood. This study aimed to characterize initial fracture response through analyzing peripheral blood hematology, multiplex cytokine profiles, and microRNA (miRNA) expression in fracture hematoma within the first 5 days post-injury. In a prospective cohort of 64 patients with acute clavicle fractures, we assessed hematological parameters, cytokine levels via multiplex immunoassays, and miRNA expression through RNA sequencing, and quantitative PCR (qPCR) validation. Fracture severity and time elapsed post-injury were key drivers of molecular response variability. Severe fractures (type C) were associated with older patient age and impaired hematological parameters, including reduced hemoglobin, erythrocyte counts, and hematocrit. Leukocyte counts declined over time, reflecting evolving systemic inflammation. Severity-dependent cytokines included eotaxin, interferon alpha-2 (IFNα2), interleukin-1 alpha (IL-1α), macrophage inflammatory protein-1 (MIP-1α), whereas interferon gamma-induced protein 10 (IP-10) and MIP-1α distinguished temporal healing phases. MiRNA profiling revealed 55 miRNAs with significant time-dependent expression changes (27 downregulated, 28 upregulated). Five key miRNAs (*miR-140-5p*, *miR-181a-5p*, *miR-214-3p*, *miR-23a-3p*, *miR-98-5p*) showed robust temporal patterns and enrichment in cytokine signaling pathways critical for bone repair. This work presents the first detailed molecular portrait of early human fracture healing, highlighting hematological, immune cytokine, and miRNA networks orchestrating repair. These insights provide a foundation for biomarkers development to predict healing outcomes and support precision-targeted interventions in fracture management.

## 1. Introduction

Fracture healing represents one of the most remarkable regenerative processes in the human body, orchestrated through four distinct, interdependent phases: inflammatory, soft callus formation, hard callus development, and remodeling. This intricate biological symphony requires precise coordination of diverse cell populations including immune cells, mesenchymal stem cells, osteoblasts, osteoclasts, and fibroblasts, driven by complex molecular mechanisms involving critical signaling pathways and transcriptional networks [1,2].

Despite this sophisticated repair machinery, disruption in the healing cascade occur in 5–10% of fractures, resulting in delayed unions or non-unions that necessitate additional surgical interventions and prolonged rehabilitation, imposing substantial clinical and economic burdens [3]. While major risk factors for impaired fracture healing have been identified, the precise molecular mechanisms underlying these complications remain incompletely understood, particularly during the critical early phases that determine healing trajectory. Elucidating these early molecular events is therefore essential for developing targeted interventions to optimize fracture outcomes [4,5].

Clavicle fractures were chosen for this study based on their unique biological, clinical, and methodological advantages. The clavicle’s subcutaneous anatomy allows minimally invasive collection of fracture hematoma during routine clinical care, facilitating temporal investigation of early healing events [6]. Clinically, clavicle fractures represent common skeletal injuries, accounting for 2.6–5% of all fractures, and exhibit well-established healing patterns that facilitate correlation of molecular signatures with clinical outcomes [7]. Importantly, clavicle fractures predominantly heal through intramembranous ossification with limited endochondral contributions, providing a relatively uniform biological model that reduces confounding variables associated with mixed healing mechanisms [8]. Furthermore, standardized surgical management protocols for displaced clavicle fractures ensure consistent and reproducible sample collection across patients [9]

The fracture hematoma, which forms immediately after injury, serves as both the structural foundation and biological command center for the initial healing response. Beyond its role as a blood clot, it functions as a reservoir of osteogenic potential, providing a fibrin scaffold for cellular recruitment and modulating the local immune microenvironment [1,10,11,12,13]. Simultaneously, systemic inflammatory response is reflected in cytokine profiles, which mirror the body’s coordinated effort to injury [14,15,16].

MicroRNAs (miRNAs) have emerged as master regulators of bone homeostasis and repair, functioning as small non-coding RNAs that regulate gene expression through targeted binding to messenger RNA (mRNA) 3′ untranslated regions. Their exquisite temporal and spatial expression patterns position them as crucial orchestrators of cell differentiation, development, and tissue regeneration [17,18,19]. Specific miRNAs have been identified as potent regulators of osteogenesis, e.g., *miR-21*, *miR-29*, or regulate osteoclast activity, e.g., *miR-31*, *miR-100*, during bone repair processes [20,21]. Additionally, circulating miRNAs have demonstrated promise as non-invasive biomarkers for monitoring bone metabolism and fracture healing progression [22]. Despite these findings, the roles of miRNAs within the human fracture hematoma during the earliest stage of healing remain poorly defined, representing a significant barrier to the development of miRNA-based diagnostics and therapies.

The insights gained from clavicle fracture healing have broad translational relevance across diverse fracture locations and clinical scenarios. The fundamental cellular and molecular mechanisms governing early inflammatory responses, hematoma organization, and initial osteogenic signaling are highly conserved across different skeletal sites, suggesting that biomarker signatures and regulatory networks identified in clavicle fractures will be applicable to other anatomical locations [5,23]. The predominantly intramembranous healing pattern observed in clavicle fractures shares key mechanistic features with critical clinical scenarios including non-union repair, distraction osteogenesis, and bone defect reconstruction, making these findings particularly relevant for developing therapeutic strategies in challenging fracture healing situations [24,25]. The establishment of standardized molecular profiling approaches in this accessible fracture model will provide a methodological framework that can be readily adapted to investigate healing mechanisms in more complex fracture types and anatomical locations [4,5].

To address this knowledge gap, we conducted comprehensive molecular analysis of early fracture healing across three distinct dimensions: peripheral blood hematological parameters, systemic cytokine expression, and miRNA landscapes within fracture hematoma during the first 5 days post-injury. We systematically examined these molecular signatures in relation to patient demographic characteristics, injury severity, and early clinical variables, with the goal of identifying key regulatory networks governing the transition from injury to repair and establishing molecular biomarkers predictive of fracture healing outcomes.

## 2. Results

### 2.1. Study Population and Design

#### 2.1.1. Study Design and Patient Characteristics

**Figure 1:**
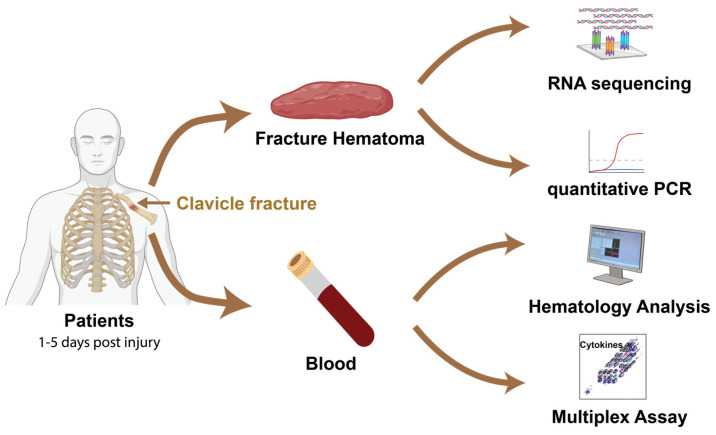
Schematic of study design and analysis workflow. Hematoma samples were harvested from patients with clavicular fractures within 1 to 5 days. Meanwhile, blood was collected to evaluate hematological parameters and cytokines profiles. miRNAs were extracted from 16 hematomas samples for RNA sequencing analysis. qPCR quantification of miRNA expression in fracture hematoma was performed on all 64 patients.

This prospective cohort study investigated early fracture healing mechanisms in 64 clavicle fractures meeting stringent inclusion criteria through comprehensive analysis of hematoma and peripheral blood samples collected within 5 days post-injury. A randomly selected subset of 16 participants underwent miRNA sequencing to identify differential expressed miRNAs across subgroups; these findings were subsequently validated in entire cohort using quantitative PCR (qPCR) of miRNAs derived from fracture hematoma. Peripheral blood was processed immediately for clinical hematology, and plasma isolated for comprehensive cytokine profiling using multiplex immunoassays. All generated molecular data were systematically correlated with fracture characteristics, patient demographics, and clinical variables to identify key factors influencing early clavicle fracture healing progression (Figure 1).

#### 2.1.2. Patient Demographics and Clinical Characteristics

The study cohort comprised 64 patients aged 18 to 80 years (median 34) with acute clavicle fractures within 1 to 5 days post-injury. To capture phase-specific molecular signatures, patients were stratified by healing stage: patients sampled within 1–3 days post-fracture (“Early”, *n* = 34, median age 37, 8 female; 11 smokers), and those sampled within 4–5 days (“Late”, *n* = 30, median age 30, 4 female; 6 smokers). The immediate post-traumatic inflammatory response occurring within days 1–3 post-injury is characterized by hematoma formation, acute immune cell infiltration, and initiation of inflammatory signaling cascades, whereas the early transitional repair phase between days 4–5 marks the beginning of cellular proliferation, tissue organization and the shift toward repair processes [26,27]. This temporal distinction is further supported by clinical evidence showing that periosteal reaction—indicating the onset of bone repair mechanisms—first becomes radiologically detectable around day 5 post-fracture [28]. Fracture severity was classified according to the standardized AO/OTA system (Type A < B < C in ascending order of complexity) [29]. Baseline hematological parameters, including hemoglobin (Hb), leukocyte count, erythrocyte count, hematocrit, and thrombocyte count, were within normal clinical ranges for all participants. Importantly, all patients demonstrated uncomplicated fracture healing during the 12-month post-operative follow-up period. Table 1 presents the baseline characteristics of all 64 patients included in the study.

### 2.2. Temporal and Severity-Dependent Hematological Changes

**Figure 2:**
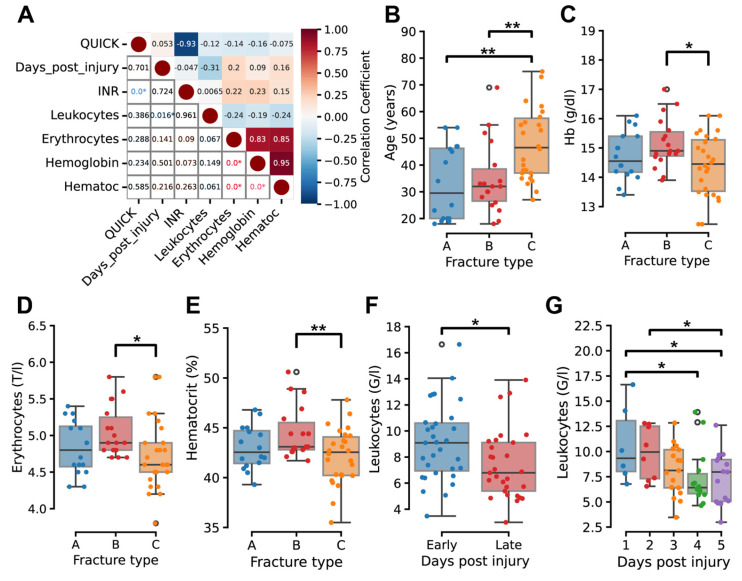
Temporal leukocyte dynamics and severity-associated hematological alterations. (**A**) Correlation matrix illustrates associations between selected numerical patient variables. Positive correlations are shown in red, negative in blue. The color gradient represents correlation strength. The upper-right triangle displays correlation coefficients, and the lower-left triangle shows corresponding *p*-values. Significant correlations (*p* < 0.05) are marked with an asterisk (*). (**B**) Age comparison among fracture types. Statistical analysis was performed using one-way ANOVA with post hoc paired comparisons. ** indicates *p* < 0.01. (**C**–**E**) Boxplots showing hemoglobin (**C**), erythrocyte count (**D**), and hematocrit (**E**) levels stratified by fracture type. One-way ANOVA with post hoc paired comparisons was used. * indicates *p* < 0.05; ** indicates *p* < 0.01. (**F**,**G**). Leukocyte levels across days post fracture injury. Student’s *t*-test and one-way ANOVA with post hoc paired comparisons were used. * indicates *p* < 0.05.

Comprehensive analysis of clinical blood parameters revealed significant temporal and severity-dependent patterns in hematological measures. Leukocyte count demonstrated a moderate, statistically significant negative correlation with days post-injury (r = −0.37, *p* < 0.05); values were significantly lower in the late phase (days 4–5) compared to the early phase (days 1–3) (Figure 2A,F,G).

Standard hematological relationships were observed within the cohort. Erythrocyte counts exhibiting strong positive correlations with both hemoglobin (r = 0.83, *p* < 0.05) and hematocrit (r = 0.85, *p* < 0.05). Prothrombin time (QUICK) and International Normalized Ratio (INR) displayed a strong inverse correlation (r = −0.93, *p* < 0.05) (Figure 2A and Figure S1A).

Analysis by fracture severity demonstrated age differences across fracture types. Patients with type C fractures had a higher mean age (45 years) than type A (30 years) and type B (32 years) (Figure 2B). Hematological parameters varied by fracture type, with type C fractures associated with significantly lower erythrocyte count, hemoglobin levels, and hematocrit compared to type B (*p* < 0.05, Figure 2C–E).

### 2.3. Temporal Dynamics of Cytokine Profiles

**Figure 3:**
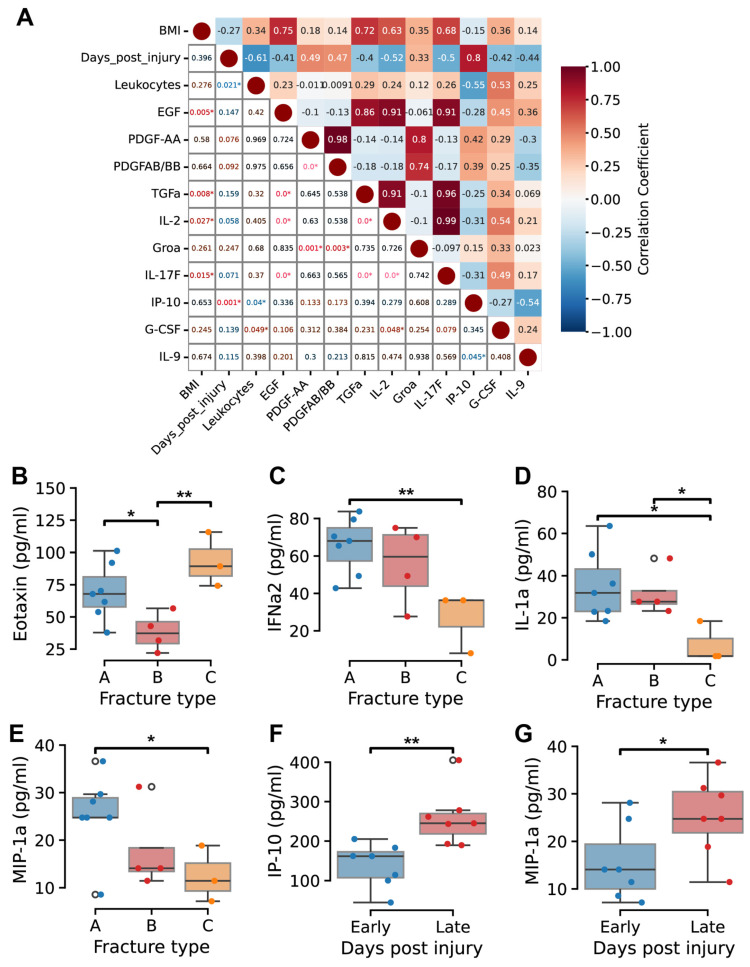
Cytokine expression patterns by fracture severity and time post-injury. (**A**) Correlation matrix showing associations between selected clinical parameters and cytokine concentrations. Positive correlations are indicated in red, while negative in blue. The color gradient represents correlation values, with the upper-right triangle displaying correlation coefficients and the lower-left triangle showing the corresponding *p*-values. Significant correlations (*p* < 0.05) are marked with an asterisk (*). (**B**–**E**) Boxplots depicted the concentrations of eotaxin (**B**), IFNα2 (**C**), IL-1α (**D**), MIP-1α (**E**) in fracture types A, B, and C. One-way ANOVA with post hoc paired comparisons were used. * indicates *p* < 0.05; ** indicates *p* < 0.01. (**F**,**G**) Concentrations of IP-10 (**F**) and MIP-1α (**G**) comparing early and late post-injury periods. Student’s *t*-test was used to compare early (days 1–3) and late (days 4–5) post-injury periods, * indicates *p* < 0.05; ** indicates *p* < 0.01.

Multiplex analysis of 48 cytokines was conducted on plasma samples collected at defined time points post-injury. Correlation analysis identified statistically significant associations among cytokines, revealing clusters of positively correlated factors (Figure 3A and Figure S2A). For example, BMI, epidermal growth factor (EGF), transforming growth factor-α (TGFα), interleukin-2 (IL-2), and IL-17F showed strong positive correlations (r = 0.63–0.99, *p* < 0.05). G-CSF exhibited moderate positive correlations with EGF, IL-2, IL-17F, and circulating leukocyte counts (r = 0.45–0.53). PDGF-AA demonstrated strong positive correlations with growth-regulated oncogene-α (GROα) (r = 0.8, *p* < 0.05), PDGF-AB/BB (r = 0.98, *p* < 0.05), and IL-4 (r = 0.72, *p* < 0.05).

Importantly, IP-10 (CXCL10) strongly correlated with days post-injury (r = 0.80, *p* < 0.05), while showing moderate negative correlations with IL-9 (r = −0.54, *p* < 0.05) and circulating leukocyte counts (r = −0.55, *p* < 0.05) (Figure 3A).

Comparative by fracture severity revealed elevated concentrations of IL-15, IP-10, and macrophage inflammatory protein-1β (MIP-1β) in type C fractures, alongside decreased levels of IFNα2, hemoglobin, hematocrit, MIP-1α, IL-1 receptor antagonist (IL-1Ra), IL-17A, EGF, and IL-9 (Figure S2B).

Eotaxin concentrations were significantly higher in type C fractures (*p* < 0.05), while IFNα2, IL-1α, and MIP-1α levels were significantly lower compared to less severe fractures (*p* < 0.05) (Figure 3B–E). Differences in PDGF-AA, IL-17A, and IL-1Ra levels between fracture types did not reach statistical significance (Figure S2B–E). Temporal analysis showed IP-10 and MIP-1α increased significantly over the post-injury period (*p* < 0.05) (Figure 3F,G). IL-4 trended upward without reaching statistical significance (Figure S2F).

### 2.4. Expression Profiles of miRNAs in Fracture Hematoma

**Figure 4:**
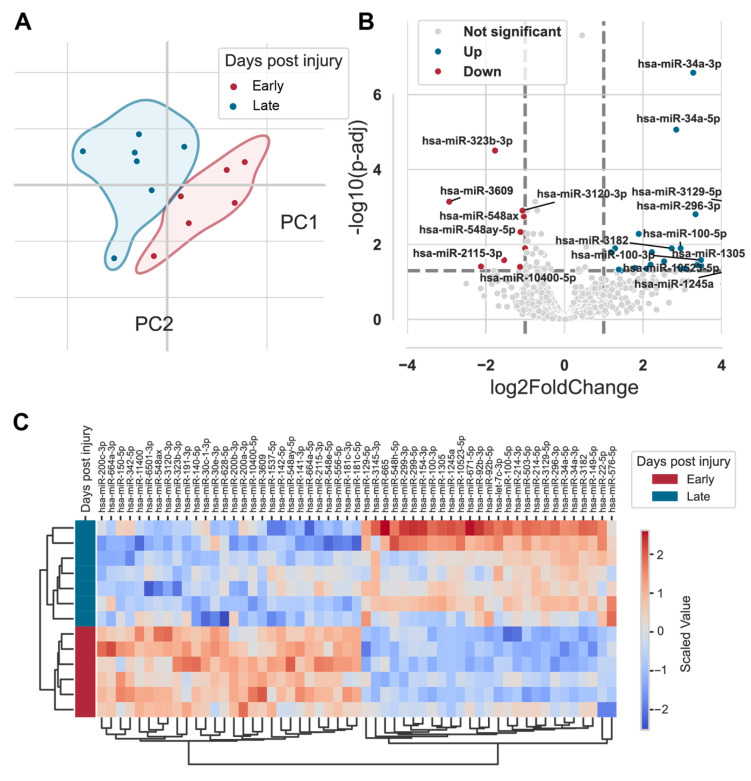
Identification of differentially expressed miRNAs in hematoma based on days post injury. (**A**) Principal component analysis (PCA) of RNA-seq data illustrating variability in miRNA expression between early (red) and late (blue) days post injury. (**B**) Volcano plot displaying significantly upregulated and downregulated miRNAs comparing early versus late days post injury. The *X*-axis represents log_2_ fold-change, and the *Y*-axis represents –log_10_ false discovery rate (FDR). Upregulated miRNAs are shown as blue dots on the right, while downregulated miRNAs are shown as red dots on the left. (**C**) Heatmap showing differentially expressed miRNAs between early and late days post injury.

Samples from 16 randomly selected patients underwent RNA-sequencing, identifying 1862 distinct miRNAs with detection across samples. Principal component analysis (PCA) revealed separation of miRNA expression profiles based on time post-injury (early vs. late), and three statistical outliers were excluded from analysis (Figure 4A).

Temporal analysis identified 55 miRNAs with statistically significant differential expression (*p* < 0.05) (Table S1). Of these, 27 miRNAs (e.g., *hsa-miR-34a-5p*, *hsa-miR-100-5p*, *hsa-miR-214-3p*) were upregulated in the late post-injury phase, while 28 miRNAs (e.g., *hsa-miR-142-5p*, *hsa-miR-30e-3p*, *hsa-miR-140-5p*) were downregulated (Figure 4B,C).

Target gene enrichment analysis using Gene Ontology (GO) revealed significant enrichment in biological processes including programmed cell death, apoptotic signaling cascades, and cytokine-mediated immune responses (Figure S3A). KEGG pathway analysis implicated neurodegenerative diseases pathways and highlighted target genes associated with long-term depression signaling and tryptophan metabolism, including *RIN2*, *TCF7L1*, *DDIT4*, and *KLF11* (Figure S3B–F).

### 2.5. Quantitative PCR Validation and Function Analysis of Key MicroRNAs

**Figure 5:**
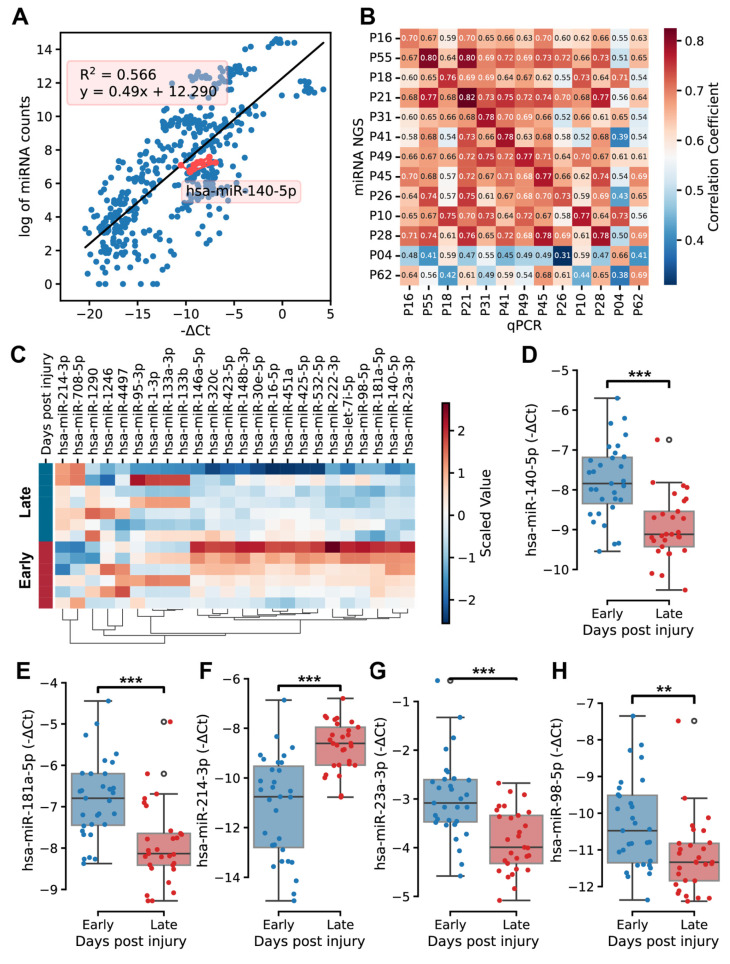
Validation of differentially expressed miRNAs in hematoma based on days post injury. (**A**) Comparison of miRNA expression levels between qPCR and next-generation sequencing (NGS). The R-squared value and regression line for *miR-140* were shown. The *X*-axis (−∆Ct) represents the difference between the Ct of the miRNA and the Ct of the reference miRNA, while the *Y*-axis indicates the log-transformed, normalized counts of miRNA. Each dot in the plot represents an individual miRNA, with red dots highlighting all *miR-140* across patients. (**B**) Heatmap displaying correlation of miRNA expression in hematoma between NGS and qPCR methods. (**C**) Heatmap of miRNAs expression validated by qPCR during early and late days post injury. (**D**–**H**) Expression profiles of validated, significantly differentially expressed miRNAs over days post injury (y = −∆Ct): *miR-140-5p* (**D**), *miR-181a-5p* (**E**), *miR-214-3p* (**F**), *miR-23a-3p* (**G**), and *miR-98-5p* (**H**). ** represents *p* < 0.01, and *** represents *p* < 0.001.

qPCR was performed to validate selected miRNAs in all 64 patients, including *miR-140-5p*. A Strong correlation was observed between miRNA expression levels obtained from RNA-seq and qPCR (R^2^ = 0.566, Figure 5A). Within individual patients, miRNA expression levels measured by both methods exhibited correlations typically greater than 0.7 (Figure 5B).

Analysis of miRNA expression profiles in qPCR data revealed different temporal profiles between the early (days 1–3) and late (days 4–5) stages of fracture healing. *miR-214* and *miR-708* showed higher expression during the late stage, while more miRNAs had higher expression during the early healing stages (Figure 5C). qPCR analysis validated differential expression of five miRNAs across days post-injury. *miR-140-5p*, *miR-181a-5p*, *miR-23a-3p*, and *miR-98-5p* showed decreased expression from early to late phases, while *miR-214-3p* showed increased expression (Figure 5D–H).

Functional enrichment analysis of these validated miRNAs identified target genes involved in DNA damage response pathways, muscle proliferation, and immune response signaling, notably cytokine-mediated interleukin pathways (Figure S4A). Reactome pathway analysis showed enrichment in immune regulation, including interleukin-4 and interleukin-13 pathways, with overlap in broader signal transduction categories (Figure S4B). IL-11RA was identified as an interacting protein in these networks (Figure S4C). Wikipathway analysis indicated enrichment in PI3K-Akt signaling and interleukins singling networks, including pathways involving IL2, IL-3, and TNFα (Figure S4D). Molecular function analysis linked target genes to BH domain binding, transcription regulatory region DNA binding, and kinase inhibitor activity (Figure S4E).

## 3. Discussion

This study provides the first comprehensive characterization of molecular signatures governing early clavicle fracture healing through systematic analysis of hematological parameters, systemic cytokine profiles, and local miRNA expression patterns. Our findings reveal coordinated temporal dynamics across these molecular domains, demonstrating that the transition from acute inflammatory response to early repair processes involves complex regulatory networks that are both time-dependent and severity-specific. These insights advance our mechanistic understanding of human fracture healing and establish a foundation for developing molecular biomarkers and targeted therapeutic interventions.

Temporal hematological response reflects inflammatory resolution as demonstrated by the observed temporal decline in leukocyte counts during the initial 5-day post-injury period, which aligns with the expected resolution of acute inflammatory responses as fracture healing progresses from the inflammatory to early repair phases. This finding corroborates previous clinical observations that peripheral leukocytosis peaks within 24–48 h post-fracture and subsequently normalizes as the acute phase response subsides [14]. The preservation of standard hematological relationships, including strong correlations between erythrocyte parameters and coagulation indices, confirms the systemic stability of patients during early healing and validates the reliability of our molecular analyses.

The age-related severity pattern, where type C fractures occurred in older patients (45 years vs. 30–32 years), is consistent with established epidemiological data showing increased fracture complexity with advancing age due to bone quality deterioration and comorbidity accumulation [7]. The hematological impairments in severe fractures—reduced erythrocyte count, hemoglobin, and hematocrit—likely reflect increased tissue damage and hemorrhage inherent to high-energy trauma.

Cytokine networks reveal coordinated inflammatory and repair signaling through distinct cytokine clusters that demonstrate the coordinated nature of inflammatory and repair responses during early fracture healing. Strong positive correlations observed between growth factors (EGF, TGFα) and inflammatory mediators (IL-2, IL-17F) support the concept of concurrent anabolic and catabolic activity, challenging traditional models of discrete healing phases [5].

The temporal upregulation of IP-10/CXCL10 represents a particularly significant finding, as this chemokine is known to orchestrate monocyte and T-cell recruitment during tissue repair processes. The concurrent negative correlation with circulating leukocyte counts (r = −0.55) suggests that IP-10 may facilitate the transition from systemic inflammatory mobilization to localized tissue repair by promoting cellular migration from peripheral circulation to the fracture site. This interpretation aligns with recent evidence demonstrating IP-10’s dual role in inflammatory resolution and tissue regeneration [30].

Severe fractures exhibited a distinctive cytokine signature marked by elevated eotaxin alongside diminished IFN-α2, IL-1α, and MIP-1α levels. This pattern indicates amplified chemotactic signaling paired with impaired antiviral and immunoregulatory responses, potentially disrupting immune homeostasis and hindering repair [5,23]. Additionally, the proinflammatory signature observed in type C fractures—characterized by elevated IL-15, IP-10, and MIP-1β alongside suppressed protective factors (IFNα2, IL-1Ra)—suggests a sustained inflammatory state that may prolong healing and elevate complication risks [31].

MicroRNA landscapes reveal dynamic post-transcriptional regulation as evidenced by the identification of 55 miRNAs with significant temporal regulation within fracture hematoma, demonstrating complex post-transcriptional control during early healing. Balanced up- and downregulation patterns underscore sophisticated, phase-specific regulatory mechanisms. Validation of key miRNAs (e.g., *miR-140-5p*) via qPCR confirms their biomarker potential. *miR-140-5p*’s consistent downregulation reflects possible involvement in intramembranous ossification beyond its known cartilage homeostasis role [20].

Functional enrichment links miRNA targets to DNA damage response, immune signaling, and cytokine-mediated pathways, especially interleukin networks. These findings suggest integrated regulation across transcriptional and post-transcriptional levels during repair.

Comparative perspectives across fracture locations and bone quality considerations indicate that while clavicle fractures provide an optimal model for intramembranous bone healing, molecular responses identified may differ in endochondral and weight-bearing bones like femur or tibia due to biomechanical loading patterns, vascular supply characteristics, and cellular microenvironments variations [23,24]. Baseline bone quality factors—such as osteoporosis and trabecular architecture—may further shape cytokine and miRNA profiles, modulating repair capacity [32]. Future work integrating bone quality assessments (e.g., DEXA, high-resolution CT) is warranted to refine biomarker applicability across patient populations [33].

Translational implications into clinical practice demonstrate that molecular signatures revealed offer promise for early clinical decision-support and precision fracture care. Integrating elevated eotaxin (CCL11) with reduced IFN-α2, IL-1α, and MIP-1α reflects an inflammatory pattern that may predict impaired healing. A multimodal biomarker panel incorporating IP-10 levels, leukocyte trajectories, and validated miRNAs (*miR-140-5p*, *miR-214-3p*, *miR-181a-5p*, *miR-23a-3p*, *miR-98-5p*) could enable robust monitoring during critical early phases. Targeted therapies may include IP-10 modulation via chemokine receptor antagonists and miRNA-based interventions (antagomiRs or mimics) focusing on osteogenic enhancement. Cytokine clustering data guide selective immunomodulation, aiming to suppress excessive IL-15 and MIP-1β in severe cases while preserving essential mediators for repair. Integration of those molecular insights with clinical risk profiling—age, fracture type, and inflammatory markers—can facilitate personalized monitoring and tailored interventions to improve outcomes.

Several limitations merit consideration in interpreting these findings. The 5-day observation window, while capturing critical early events, does not encompass the complete inflammatory-to-repair transition, which may extend through the first 1–2 weeks post-injury. The focus on clavicle fractures, while methodologically advantageous, limits direct extrapolation to fractures involving significant endochondral ossification components or different biomechanical environments. The sample size for RNA sequencing (*n* = 16), though adequate for discovery-phase miRNA profiling, may have limited power to detect subtle expression changes. Future studies should incorporate larger sequencing cohorts, diverse fracture sites, longer observation periods, and bone quality stratification to enhance clinical applicability and biomarker robustness.

## 4. Materials and Methods

### 4.1. Study Population

A total of 64 patients with closed clavicle fractures admitted to the Department of Trauma Surgery at Klinikum rechts der Isar, Technical University of Munich, were enrolled. Inclusion criteria were: (I) age ≥ 18 years, (II) clavicle fracture requiring open reduction and internal fixation (ORIF), and (III) absence of pathological fractures. Patients underwent surgery 3 ± 2 days post-trauma. Exclusion criteria included pregnancy, incarceration, sepsis, HIV, hepatitis, meningitis, primary neurological or psychiatrically diseases. All patients provided written informed consent before sample collection. The study protocol was approved by the local institutional review board (Protocol No. 354/20). Relevant clinical data were extracted from medical records. Detailed patient characteristics are summarized in Table 1.

### 4.2. Sample Processing

Per patient, 9 mL of EDTA anticoagulated blood was collected during routine clinical laboratory procedure for hematological analyses and plasma isolation. Hematoma samples were collected intraoperatively using a standardized, atraumatic technique and immediately transported on ice for lab processing. Hematoma preparation followed QIAGEN manufacturer protocols (QIAGEN, Hilden, Germany). Briefly, erythrocytes were lysed by adding EL-Buffer (QIAGEN, Hilden, Germany) at a 5:1 ratio to the hematoma sample, followed by incubation and homogenization through a cell strainer. For RNA isolation, the homogenized sample was lysed with RLT Buffer (QIAGEN, Hilden, Germany) and transferred to a QIAshredder (QIAGEN, Hilden, Germany). Samples were stored at −80 °C until further analysis.

### 4.3. MiRNA Isolation and Sequencing

Total RNA was isolated from hematoma samples using the miRNeasy Serum/Plasma Kit (QIAGEN, Hilden, Germany) according to the manufacturer’s instructions. RNA concentration was measured using the Nanodrop Spectrophotometer 2000 (Thermo Fisher Scientific, Waltham, MA, USA). Small RNA libraries were prepared with the QIAseq miRNA Library Kit (QIAGEN, Hilden, Germany) and their quality verified by capillary electrophoresis on an Agilent TapeStation D1000 system (Agilent Technologie, Santa Clara, CA, USA). Libraries were pooled and sequenced on an Illumina NextSeq 2000 platform (Illumina, Inc., San Diego, CA, USA).

### 4.4. Quantitative PCR

Quantitative PCR (qPCR) was performed by Qiagen genomic services (QIAGEN, Hilden, Germany). RNA spike-in controls (UniSp2, UniSp4 for RNA isolation control; UniSp6 for cDNA synthesis) were included to monitor assay quality. Isolated RNA was reverse transcribed to cDNA and analyzed using the miRCURY LNA miRNA PCR Custom Panel (QIAGEN, Hilden, Germany) on a LightCycler^®^ 480 Real-Time PCR System in 384-well plates (Roche, Basel, Switzerland).

### 4.5. Cytokine Profiles

Plasma cytokine concentrations were measured using a 48-plex multiplex immunoassay kit HCYTA-60K-PX48 (Merck Millipore, Darmstadt, Germany) run on a MAGPIX Luminex platform (Luminex Corporation, Austin, TX, USA). Briefly, samples (25 µL) were incubated overnight with bead-based multiplex assays according to manufacturer’s protocols. Cytokine concentrations were calculated using a five-parameter logistic regression model from replicated standard curves.

### 4.6. Data Analyses

Raw sequencing data were processed with CLC Genomics Server (v.21.0.4, QIAGEN, Hilden, Germany) mapped to the human miRbase v22 reference genome. miRNA expression levels were quantified as counts per million (CPM). Calibration with RNA spike-in controls was performed for quality normalization. Differential expression analysis was carried out with PyDESeq2 (v.0.4.10; Python package), adjusting for covariates including fracture type and days post injury). DESeq2 reports a value called ‘base-Mean’ corresponding to the average of normalized count values over all samples, dividing by design factors from each covariate, with significant miRNAs identified at a false discovery rate (FDR) < 0.05. Statistical analyses were performed using SPSS Statistics v27.0 (IBM Corp, Armonk, NY, USA). Quantitative data were reported as mean ± standard deviation (SD); categorial data as percentages. Spearman’s rank correlation was used for correlation analyses. Student’s *t*-test was applied for paired comparison, while one-way ANOVA with post hoc tests was used for multiple group comparisons.

## 5. Conclusions

This comprehensive analysis establishes that early clavicle fracture healing involves coordinated molecular networks encompassing systemic hematological responses, inflammatory cytokine signaling, and local miRNA regulation. The temporal dynamics observed across these molecular domains support a model of synchronized healing responses rather than sequential phase transitions. These findings provide new molecular targets for therapeutic intervention and establish biomarker candidates for clinical monitoring of fracture healing progression. The translational potential of these molecular signatures, when integrated into clinical algorithms and adapted for diverse fracture types and bone quality states, offers promising avenues for advancing precision medicine approaches in orthopedic trauma care.

## Figures and Tables

**Table 1:** Baseline characteristics of patients (*n* = 64).

Days Post Injury	Early (*n* = 34)	Late (*n* = 30)
Age (median (min–max))	37 (18–75)	30 (18–80)
Gender	♂ 26, ♀ 8	♂ 26, ♀ 4
BMI (Mean ± SD)	23.44 ± 2.95	24.38 ± 3.63
Smoking	11 Yes	6 Yes
Type of fracture		
A	10 (29.41%)	10 (33.33%)
B	9 (26.47%)	9 (30.00%)
C	15 (44.12%)	11 (36.67%)
Hb (Mean ± SD, g/dL)	14.41 ± 1.18	14.74 ± 1.34
Leukocytes (Mean ± SD, g/L)	9.08 ± 2.99	7.28 ± 2.66
Erythrocytes (Mean ± SD, T/l)	5.04 ± 1.98	4.9 ± 0.52
Haematocrit (Mean ± SD, %)	42.19 ± 3.22	43.21 ± 3.96
Thrombocytes (Mean ± SD, G/l)	228.62 ± 47.23	225.27 ± 49.63

## Data Availability

The sequence data generated in the current study will be made available upon acceptance in public.

## Supplementary Materials

The following supporting information can be downloaded at https://www.mdpi.com/article/10.3390/ijms26188825/s1.
