# Supplementary material for: Temporal Molecular Signatures of Early Human Clavicle Fracture Healing: Characterization of Hematological, Cytokine, and miRNA Profiles
Source: Int J Mol Sci. 2025 Sep 10;26(18):8825. doi: 10.3390/ijms26188825 (PMC12469629; doi:10.3390/ijms26188825)
Supplement: Supplementary file 1 [file ijms-26-08825-s001.zip › ijms-3841266-supplementary.pdf]

Figure S1

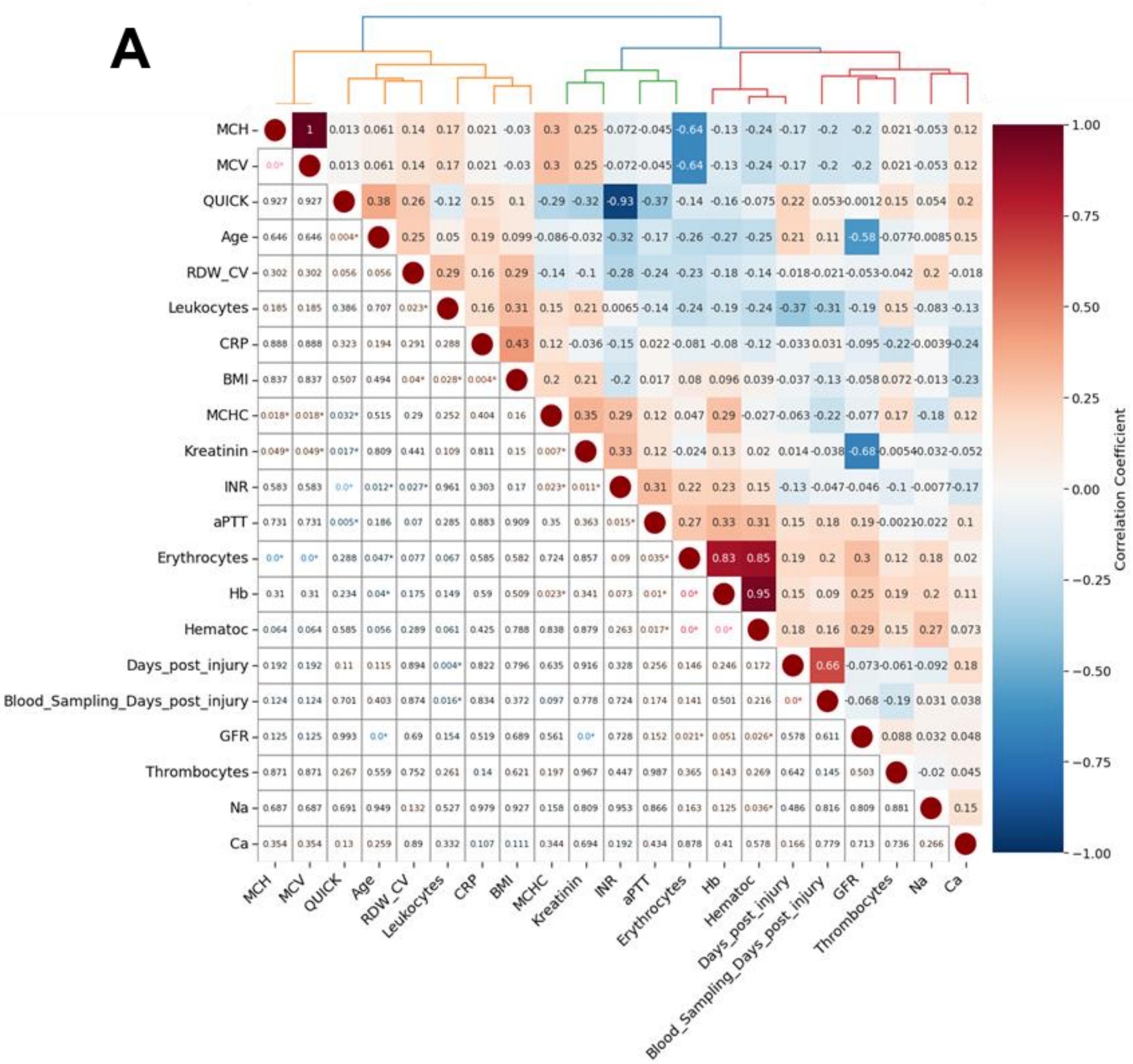

Figure S1. Comprehensive correlation matrix of patient numerical variables. (**A**) The correlation matrix illustrates the associations between various numerical patient variables. Positive correlations are indicated in red, while negative correlations are shown in blue. The colour gradient represents correlation strengths. The upper-right triangle displays correlation coefficients, and the lower-left triangle shows corresponding *p*-values. Significant correlations ($p < 0.05$) are marked with *. Tree length was calculated by using Ward method.

Figure S2

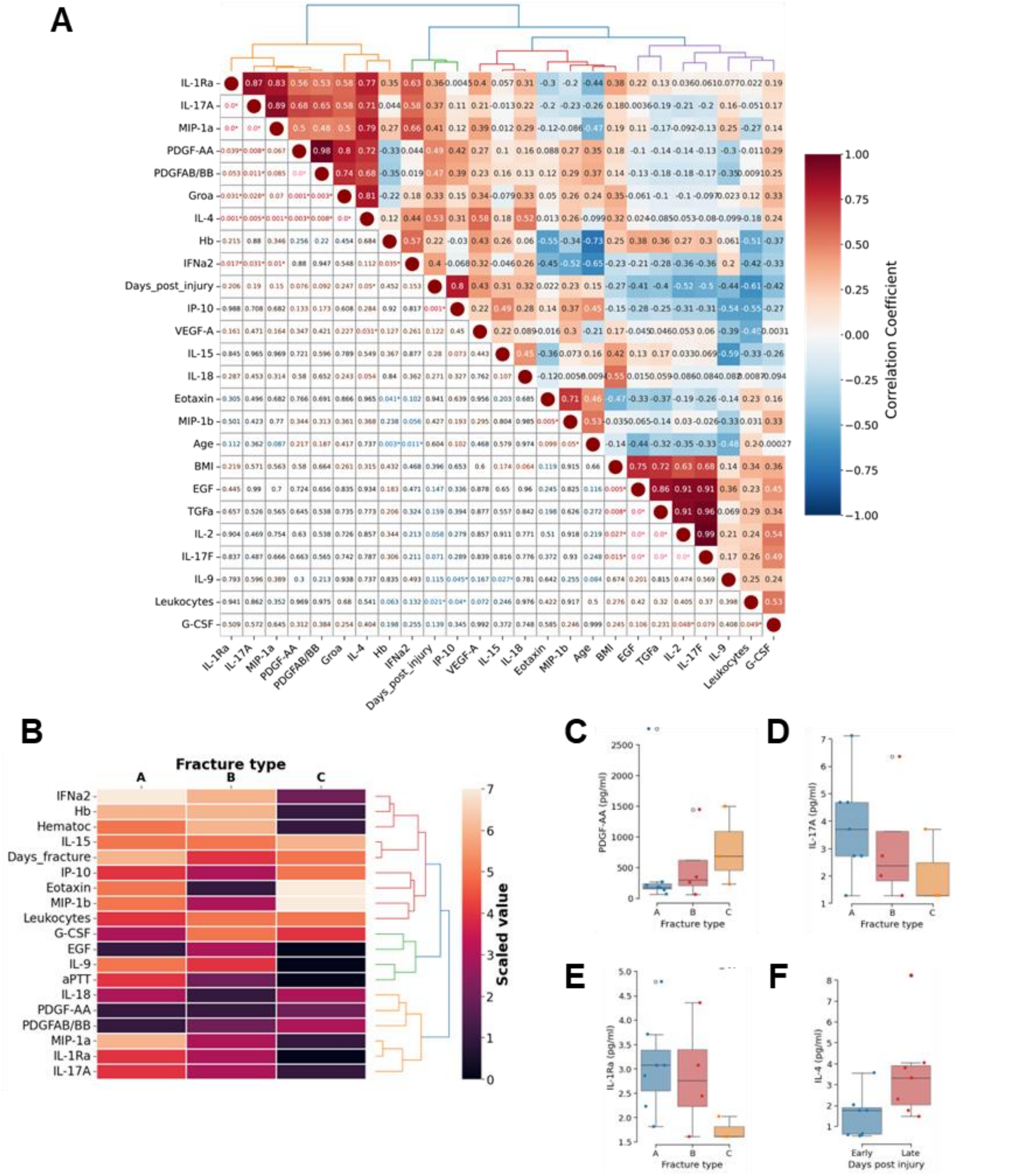

Figure S2. Cytokine expression patterns by fracture severity and time post-injury. (**A**) Correlation matrix revealed association between clinical parameters and cytokine concentrations. Positive correlations are indicated in red, while negative in blue. The colour gradient represents correlation values, with the upper-right triangle displaying correlation coefficients and the lower-left triangle showing the corresponding *p*-values. Significant correlations, *p*-value < 0.05, are marked with *. Tree length was calculated by using Ward method. (**B**) Scaled cytokine levels were presented based on fracture type A, B, C. The scaled values range from 0 (dark red) to 7 (light orange), representing expression levels from weak to strong. Tree length was calculated using Ward methods. (**C-E**) Boxplots depicted the concentration of PDGF-AA (C), IL-17A (D), and IL-1Ra (E) in fracture types A, B, and C. (**F**) Boxplots depicted the concentration of IL- 4 between the early and late post-injury periods.

Figure S3

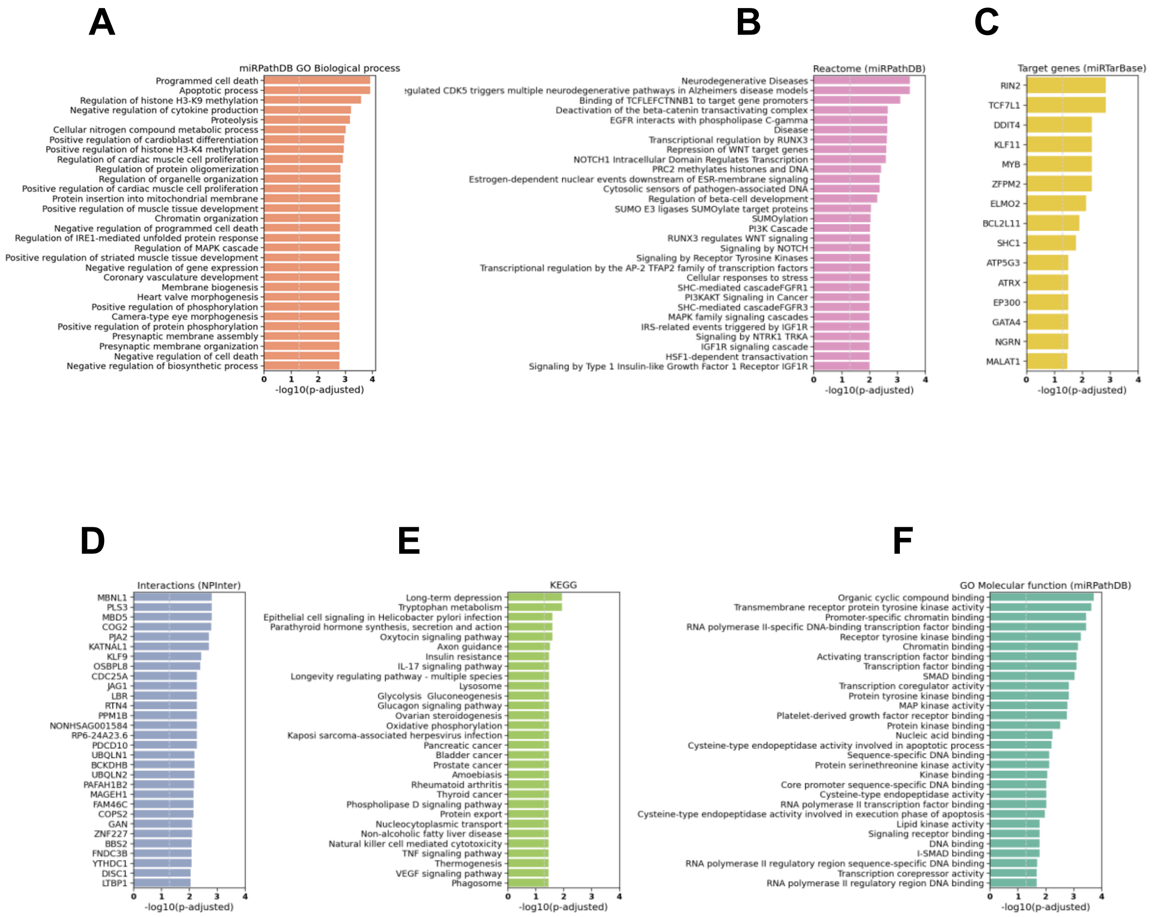

Figure S3. Functional enrichment analysis of differentially expressed miRNAs over days post-injury. (A) Top GO biological processes annotated by miRPathDB. (B) Reactome pathways of miRNA target genes identified by miRPathDB. (C) Predicted target genes based on differentially expressed miRNAs. (D) Top interacted proteins predicted by NPInter. (E) Top enriched KEGG pathways. (F) Top GO molecular functions annotated.

Figure S4

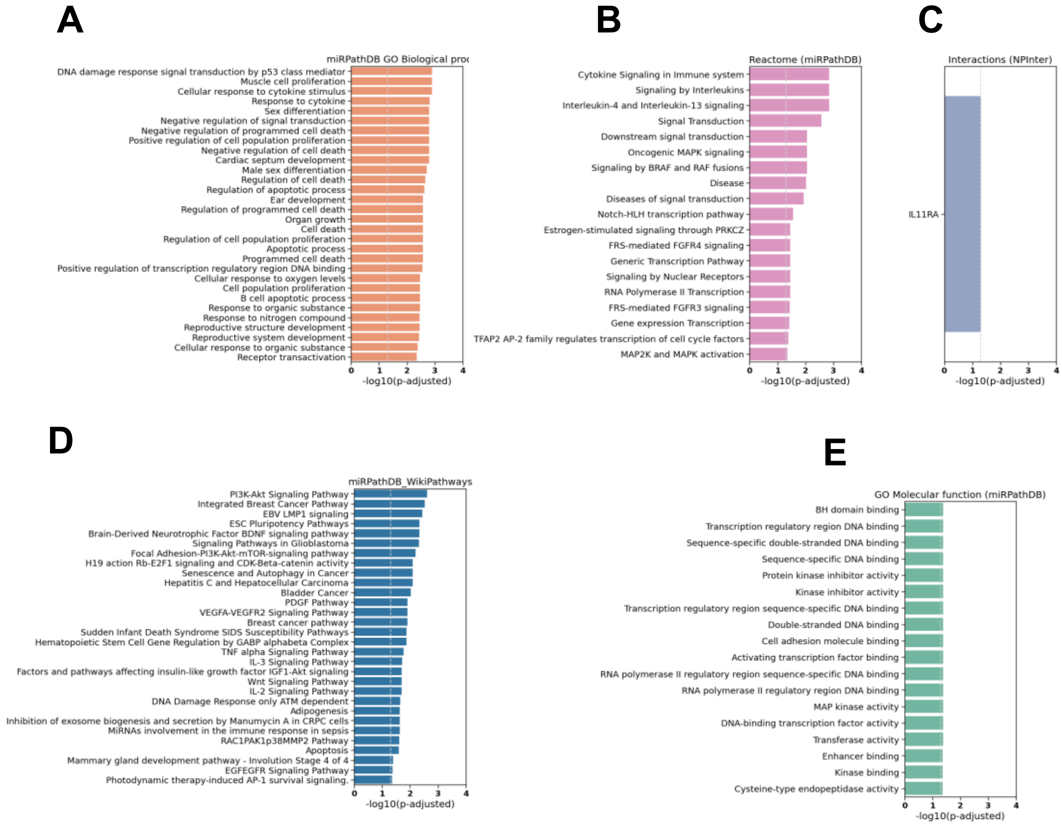

Figure S4. Functional enrichment analysis of validated differentially expressed miRNAs from qPCR data over days post-injury. (A)Top Gene Ontology biological processes annotated via miRpathDB analysis. (B) Top Reactome pathways identified via miRPathDB. (C) Top interacting protein predicted. (D) Top Wikipathways identified via miRPathDB. (E) Top Gene Ontology molecular functions annotated.

Table S1

Seq_id	baseMean	log2FoldChange	lfcSE	stat	pvalue	padj
hsa-miR-142-5p	267921.2086	-0.582637553	0.194626354	-2.993621065	0.002756881	0.044356248
hsa-miR-30e-3p	26489.87134	-0.505870686	0.168845404	-2.996058363	0.002734941	0.044356248
hsa-miR-34a-5p	10564.29006	2.84576493	0.512847265	5.548952144	2.87387E-08	8.63118E-06
hsa-miR-150-5p	12471.48985	-0.958786593	0.303923991	-3.154692031	0.001606676	0.035307686
hsa-miR-140-5p	1230.439946	-0.590932156	0.1501726	-3.935019803	8.31898E-05	0.005208127
hsa-miR-100-5p	2794.248275	2.956557328	0.820852629	3.601812581	0.000316006	0.012669351
hsa-miR-671-5p	914.4139763	1.43696363	0.48799706	2.944615344	0.003233564	0.049380358
hsa-miR-22-5p	583.893829	0.556000082	0.151539963	3.66899972	0.000243501	0.01154709
hsa-miR-503-5p	554.7635937	1.288365846	0.356283822	3.616122219	0.000299049	0.012669351
hsa-miR-141-3p	485.8838503	-0.690950185	0.158200544	-4.367558833	1.25643E-05	0.00124688
hsa-miR-576-5p	526.9830071	0.525367996	0.171476076	3.063797627	0.002185467	0.040185831
hsa-miR-3182	403.0786187	2.728811656	0.757916712	3.600410983	0.000317715	0.012669351
hsa-miR-342-5p	423.6550732	-0.927566685	0.237695581	-3.902330375	9.5271E-05	0.005208127
hsa-miR-181c-3p	325.1155848	-0.78934481	0.202664627	-3.894832666	9.82665E-05	0.005208127
hsa-miR-299-3p	511.64049	2.225191456	0.635597348	3.500945157	0.000463611	0.016065913
hsa-miR-664a-5p	250.7291977	-0.507856222	0.149423496	-3.398770849	0.000676894	0.019058798
hsa-miR-30c-1-3p	290.3876689	-0.727627685	0.202710388	-3.589493817	0.000331321	0.012669351
hsa-miR-323b-3p	625.9746904	-1.766633419	0.335397472	-5.267283046	1.38458E-07	3.11876E-05
hsa-miR-200c-3p	289.0119019	-0.744389369	0.163454104	-4.554118575	5.26057E-06	0.000721887
hsa-miR-556-5p	190.3458358	-0.959703684	0.220768517	-4.347103904	1.37947E-05	0.00124688
hsa-miR-628-5p	193.7435144	-0.72384313	0.233515833	-3.099760397	0.001936772	0.038778489
hsa-miR-11400	210.7098793	-1.001678099	0.279432013	-3.584693419	0.000337474	0.012669351
hsa-miR-181c-5p	155.6483557	-0.566550696	0.163803283	-3.458726132	0.000542736	0.01660287
hsa-miR-214-3p	191.1670135	3.060702968	1.018772497	3.004304667	0.002661886	0.044356248
hsa-miR-191-3p	139.515846	-0.600621769	0.173156737	-3.468659539	0.000523062	0.01660287
hsa-miR-548ax	125.6782814	-1.034034896	0.244598475	-4.227478923	2.36324E-05	0.001774402
hsa-miR-3120-3p	117.8643825	-1.067005519	0.245491645	-4.346402571	1.38388E-05	0.00124688
hsa-miR-299-5p	226.4848682	2.536164981	0.778711154	3.256875117	0.00112646	0.028192786
hsa-miR-92b-3p	132.5732819	1.890963344	0.48481372	3.900391568	9.60372E-05	0.005208127
hsa-miR-2115-3p	76.83295333	-1.536718949	0.466900145	-3.291322494	0.000997175	0.026046283
hsa-miR-34a-3p	32.64474044	3.277857645	0.529040891	6.195849323	5.79716E-10	5.22324E-07

**hsa-miR-548ay-5p**	53.49129847	-1.118159165	0.280436641	-3.987207803	6.68555E-05	0.004633598
**hsa-miR-664a-3p**	56.07185183	-0.704016419	0.217660609	-3.234468667	0.001218693	0.02967682
**hsa-miR-149-5p**	103.8215586	3.480006269	1.112071046	3.129302109	0.001752221	0.036715132
**hsa-miR-548e-5p**	43.93459173	-0.814532665	0.237760282	-3.425856741	0.000612864	0.017812582
**hsa-miR-200a-3p**	47.01390088	-0.929436675	0.246321312	-3.773269425	0.000161122	0.008065058
**hsa-miR-92b-5p**	37.63877373	1.186270668	0.338388782	3.505644188	0.000455504	0.016065913
**hsa-miR-6501-3p**	19.57658596	-0.958485913	0.275671888	-3.476908431	0.000507231	0.01660287
**hsa-miR-665**	43.88084063	2.084551741	0.692719776	3.009227994	0.002619125	0.044356248
**hsa-miR-154-3p**	60.35699404	2.192128264	0.692018577	3.167730372	0.001536339	0.034606044
**hsa-miR-214-5p**	11.94459813	3.381196814	1.057593483	3.197066611	0.001388329	0.032918002
**hsa-miR-200b-3p**	15.93035724	-0.887793217	0.288148397	-3.081027785	0.002062874	0.039545733
**hsa-miR-1537-5p**	11.9724897	-1.131141325	0.36634263	-3.087659568	0.002017394	0.039514615
**hsa-miR-296-3p**	15.68230941	3.336141081	0.780188692	4.276069516	1.90222E-05	0.00155809
**hsa-miR-3129-5p**	25.42672139	4.146766605	0.913256507	4.540637348	5.60844E-06	0.000721887
**hsa-miR-129-5p**	16.04068883	1.3834277	0.466188557	2.967528224	0.003002047	0.04663525
**hsa-let-7c-3p**	3.99197674	2.902965489	0.962730746	3.015345152	0.00256687	0.044356248
**hsa-miR-548h-5p**	10.0589803	1.79776272	0.590807779	3.042889389	0.002343184	0.042224184
**hsa-miR-100-3p**	13.10889845	3.474039112	1.055009199	3.292899357	0.0009916	0.026046283
**hsa-miR-3145-3p**	2.827010101	3.377704495	1.073886639	3.145308239	0.001659119	0.035592058
**hsa-miR-1245a**	3.903104746	5.385195762	1.638217629	3.287228551	0.001011787	0.026046283
**hsa-miR-10400-5p**	3.50649823	-2.124227786	0.684390915	-3.103822305	0.00191038	0.038778489
**hsa-miR-10523-5p**	3.178067021	4.065101535	1.324083994	3.070123613	0.002139702	0.040163988
**hsa-miR-3609**	7.298540706	-2.93298041	0.643440118	-4.55828029	5.15742E-06	0.000721887
**hsa-miR-1305**	5.45523076	4.87653453	1.411946584	3.453767009	0.000552815	0.01660287
